# A Narrative Review of Stigma Related to Infectious Disease Outbreaks: What Can Be Learned in the Face of the Covid-19 Pandemic?

**DOI:** 10.3389/fpsyt.2020.565919

**Published:** 2020-12-02

**Authors:** Fahimeh Saeed, Ronak Mihan, S. Zeinab Mousavi, Renate LEP Reniers, Fatemeh Sadat Bateni, Rosa Alikhani, S. Bentolhoda Mousavi

**Affiliations:** ^1^Department of Psychiatry, Psychosis Research Center, University of Social Welfare and Rehabilitation Sciences, Tehran, Iran; ^2^Department of Psychology, Washington State University, Pullman, WA, United States; ^3^Institute of Clinical Sciences & Institute for Mental Health, University of Birmingham, Birmingham, United Kingdom

**Keywords:** corona virus disease (COVID-19), stigma & discrimination, health consequence, pandemics, mental health, public health

## Abstract

Infectious disease pandemics are associated with social consequences and stigma that are noticeably similar in various health conditions, health systems, and cultures. Stigma impacts health-related outcomes, not only as a barrier to receiving the timely diagnosis and appropriate treatment but also as an important variable that increases mental health issues such as anxiety and depression. The COVID-19 outbreak has been associated with stigma too. Studying similarities as well as differences in the features of stigma observed in each outbreak can provide us with the knowledge and deeper understanding of the situation, which is necessary for approaching the issue comprehensively. The stigma needs to be addressed rigorously by professionals and health care providers as well as authorities. Here, we narratively review stigma due to some well-known infectious diseases and how it parallels to the current COVID-19 situation. After discussing its effects on both individuals and societies, we provide solutions to manage this important issue.

## Introduction

The Covid-19 pandemic is progressively known as a social problem rather than just an infectious disease. There are diseases that not only burden the medical system but also by provoking social stigma, lay an increased tension on each individual. Contagious diseases such as acquired immunodeficiency syndrome (AIDS), Ebola, some types of severe Influenza such as H1N1 and COVID-19 are among those (1–3). They seem to be mysterious due to a lack of sufficient knowledge about them and being lethal (4, 5). Nowadays, many people suffering from COVID-19 are challenged doubly, both by the disease and by the stigma associated with it.

Stigma has been defined as an identifying mark of disgrace or one defining characteristic that is related to a particular context, quality, or person. It is a deleterious label that makes the stigmatized person or group feel secluded from mainstream society (6, 7). Labeling can further develop into pigeonholing and stereotype formation, therefore leading to discrimination and status loss (8). In the case of stigma related to infectious diseases, stigmatization, blaming others, and discrimination are exacerbated by fear of illness. It is not always done consciously and might serve several means. Firstly, in the face of an unfamiliar or intolerable adverse situation (here, lack of knowledge about the source of infection and protective measures) or an unknown hazard (insufficient scientific understanding about the infection source), stigmatization can temporarily bring feelings of security even if it is not a real or a permanent one. This is done by distancing from the source of the threat (here infected people), through dividing people into “them” and “us.” The underlying thought is: we are not them, we do not possess the same risk factors, so we are not in danger (1). Individuals may be the victims of persistent discriminatory behavior and prejudice in such situations. Secondly, attributing a familiar cause—even if it is not a real or relevant one- changes an unknown situation to a more recognized one. Finding a culprit for a disease changes a mysterious and scary disease into a more tangible and controllable one. The anger caused by the disease can be directed toward the attributed source and by reducing the feeling of responsibility for spreading the disease, it can temporarily reduce anxiety. In the case of infectious disease outbreaks, scapegoating and projecting negative emotions onto a group of people, attributing the cause of their illness to their irresponsibility or their poor morals or even their prior health conditions are some examples (1, 4, 5).

Stigma has several features. Self-stigma happens when the stigma is internalized, so it affects attitudes, emotions, and even beliefs of individuals and forms the behavior of the people who are stigmatized (9). Internalized stigma can induce the feeling of inferiority and rage turned inward (10). Stigma interferes with the process of diagnosis and treatment by disrupting social communication, individual identity, and the sense of free will (11). People who feel stigmatized are susceptible to avoiding certain behaviors that they feel might increase stigma; in the case of COVID-19 that can be getting tested, because a positive test can be the label they consider stigmatizing. Consequently, by preventing social adjustment and healthy adaptive behaviors, stigmatization can exacerbate physical health problems (10). People who have internalized stigma, are less likely to follow health guidelines provided to control infections (e.g., wearing face covering, keeping distance from others and not mixing with other households), have less tendency to undergo diagnostic tests or comply with test-and-trace systems, and even are reluctant to receive necessary treatments (11, 12). Stigma, therefore, can be a barrier to effective prevention and control mechanisms and can affect, not only the stigmatized group, but also a wide range of people, including patients, families, friends, and the whole community.

Perceived stigma is another important facet of stigma; that is, how much people expect stigma from society and even health care providers. In most infectious diseases, perceived stigma is high and is associated with health-related outcomes (13, 14). Even the decision of whether to seek help from traditional healers or the conventional health care system is related to perceived stigma (15, 16). Literature suggests that in many cultures such as Africans and Asians, stigma is associated with approaching traditional healers as a first step (16, 17). This will further complicate the situation by delaying accurate diagnosis and treatment. Perceived stigma not only discourages infected individuals from accessing needed health care services (18) but it also affects health care professionals themselves (3, 19). In the case of COVID-19, for instance, stigma has highly influenced health care workers' performance by increasing fatigue, burnout, and decreasing satisfaction. Perceived stigma and discrimination also affect health care providers' sense of self-efficacy, and increase psychological distress and somatic symptoms (3).

Although there are arguments suggesting that the stigma related to the disease might have some evolutionary functions, that is, avoiding the source of infection, and staying safe by distancing from people who are infected with the disease (20), the costs of stigmatization almost always outweigh the hypothetical benefits (1, 10). There seems to be a delicate line separating stigmatization from necessary infection prevention measures that encourage social distancing and avoiding the source of contamination. Avoiding the source of hazard prudently can reduce the hazard but stigmatization has a component of moral judgment about an individual, a group of people, or a place (21). People and infection must not get conflated. Avoiding the source of infection and disease must clearly get distinguished from the whole character of an individual, their ethnicity, cultural, religious and socioeconomic background, and place of living; otherwise, the consequences can be serious as will be discussed below.

Stigma is a pressing issue and its consequences are noticeably similar in various health conditions, health systems, and cultures (22, 23). Here, we evaluate stigma related to some well-known infectious diseases and how it parallels to the current COVID-19 situation. Then, we review its effects on both individuals and societies and how disease-related fear and impulsive measures can be generalized and projected to irrelevant features such as ethnic background or the place of living. Consequences of stigma on public health and individuals' life as well as some solutions are provided.

## Stigma in Previous Epidemics and Pandemics

Throughout history, human beings have been exposed to dangerous diseases that have forced them to modify their behavior to adapt to new conditions. The WHO has defined a pandemic as “a worldwide spread of a new disease” (24). From the 19th century's smallpox to the 21st century's COVID-19, epidemics and pandemics have always been associated with stigma and severe social consequences (25–27). Dealing with several outbreaks delivers a wealth of knowledge on what the consequences of stigmatization are in society and how to deal with them effectively. Despite similarities, each infectious disease outbreak, such as plague, tuberculosis, syphilis, HIV, and hepatitis have represented different features of stigma that should be addressed to plan preventing or eliminating stigma measures scientifically (1, 10, 28). These differences could explain why we struggle with stigma so much, even though we have been through pandemics before.

Stigma exists in a variety of cultures and its consequences are markedly parallel in various health conditions, health systems, and cultures (22). In almost all pandemics, minorities [whether due to ethnicity (5), sexual orientation (11), gender identity (29), or place of living (2)] are at the highest risk of stigmatization. Both fears of exposure to illness and fear of dissimilarity (here people who we think are different) result in stigma (2, 21, 30). In all outbreaks, insufficient knowledge about the prognosis and outcomes, how long it could take until a cure is found and no availability of an effective treatment option or vaccine for prevention, are the main sources of fear (26). There are, however, differences in the features of stigmatization in each pandemic that can color our understanding of stigma. These differences present important areas of investigation and can be categorized as below:

### Risk Appraisal

The difference in estimated risk and related response is related to stigma (21, 23). Even when estimated risk is low, stigmatization happens. There are, for example, several reports of Americans stigmatizing Africans and their neighborhoods during the Ebola outbreak in West Africa despite the risk to Americans being declared low (2, 21). Sending conflicting messages from media has been suggested to play a role in this (21). In the Covid-19 pandemic, the risk is high for almost all countries. This can increase fear which can result in increased stigmatization toward people who are assumed to be infected. By distortions of hazard appraisal, social stigma can lead to anxiety and panic in society and can even affect the distribution of resources by authorities (23, 31). For example, government regulated distribution of personal protective equipment (PPE) as well as its shortages gave high levels of conflict and uncertainty as hospitals were prioritized whereas people working in care homes had to work under close contact with those affected but without proper PPE available to them.

### Transmission Way

Respiratory viruses are more contagious than other well-known stigmatizing diseases and there is less control on its spreading; therefore, the social reactions can be dissimilar. Fear of a global pandemic combined with scenarios of economic breakdowns and shortages can exacerbate fear driven reactions along with stigma in these cases (5, 19, 23).

### Lethality

Lethality of a disease is one of the factors that causes stigmatization. On the other hand, most of these viruses are less lethal than some sexually transmitted diseases (STDs) such as HIV. The contagion of Covid-19 is much higher, which introduces new challenges. In the case of COVID-19, people opposing the measures to stop the rate of infection state that COVID-19 is about as lethal as the flu. However, with COVID-19 being much more contagious, death rates soar if nothing is being done.

### Difference in the Population Who Are at Risk

Prejudice and established social norms are associated with stigma (1, 4). For example, in STDs such as HIV, homosexual men have been (and continue to be although to a lesser extent) stigmatized, which contributed to less funding on research and delaying treatment in the 1980s (1, 30). In respiratory viruses such as MERS and Covid-19, the main at-risk groups are the elderly, people with immune deficiencies, and individuals suffering from cardiopulmonary illnesses (19). These groups often face different forms of stigma that will be discussed later.

## Risk Factors and Vulnerable Groups

### Ethnic Background and Place of Living

Established social hierarchies and some biases in attitudes regarding ethnic minorities and immigrants predispose them to risk of stigmatization (2, 4, 5). Phenotypic features such as the color of the skin and even the accent of speech can be a source of stigmatization (2). When an association of the disease takes place with a particular ethnic background or even a place of living or origin, it might shape a metonymy that completely associates the disease with that particular group or place. For instance, in the nineteenth century, after several smallpox epidemics in Chinatown in San Francisco, the metonymy of the infection and the region took place (25). This association caused severe racialism and xenophobia during that period. During H1N1 epidemics in the US, the Mexicans and immigrants from South America were considered the main population that contributed to spreading the disease and therefore faced severe stigma (5). Currently, as COVID-19 started in Wuhan, associating it with the originating place (China) resulted in labels such as “Chinese Virus” or “Wu Flu.” One study found that there has been a prominent rise in application of the terms “Chinese virus” or “China virus” on Twitter in the USA (32). This goes strongly against the recommendations of the World Health Organization (WHO), who recommends avoiding the use of geographic locations for naming a disease as a practical way to minimize its unnecessary negative impact. Reports of people with Asian phenotype who have been victims of racist attacks on several occasions is proof of stigma and discrimination that could have been avoided (32, 33). Naming diseases such as “swine influenza” and Middle Eastern Respiratory Syndrome (MERS) has been harmful due to generating stigma and affecting societies, tourism, industries, and economics (34).

Ethnic minorities often get affected disproportionately in pandemics such as COVID-19 (35, 36). They might be at greater risk of infection due to several socioeconomic factors and inequalities in access to health care; where and in what circumstances they live, their access to good nutrition and legitimate information, their job situation that may necessitate social contact or increase potential exposure, as well as lack of paid sick leave and having hourly jobs. However, increased discrimination against them in almost every pandemic has been documented (1, 5, 25). In the US, Blacks, Asians, and minorities (BAME individuals) have been seriously affected by COVID-19 and experience a disproportionate number of deaths. Along with previously mentioned reasons, stigma, discrimination, and health disparities have been suggested to play a crucial role (36, 37).

### Double Stigma

People who have experienced stigma (whether due to a chronic health condition or other factors such as their sexual orientation or aging) are at the risk of experiencing double stigma (38, 39). In many countries, COVID-19 was first introduced as a disease that mainly affects the elderly or people with chronic health conditions. This was intended to prevent the panic that an epidemy might cause in the society but it backfired as emphasizing it on many occasions caused severe distress in the mentioned groups (11, 13). Here we see the subtle line between providing accurate and scientific information and overemphasizing that leads to misguidance and feelings of insecurity in society. Persistently opposing the “general public” to “at-risk group” carries out the message of “in-group” vs. “out-group” that contributes to stigma formation (11). Stigma makes vulnerable people more susceptible, as altering unhealthy behavior becomes even more challenging; it can also shift an extra burden toward them while directly affecting their mental health (26).

Aging has been associated with stigma and stereotypes in several cultures (40, 41). In many western countries, attitudes toward older adults are predominantly negative (42). The stigmatizing perspective is mostly related to being weak or non-productive in a society that overvalues being young, healthy, and productive (40, 42). The elderly are at risk of emotional distress by the messages they get from society. Self-Image, perceptions, and beliefs are strong moderators for age-related stigmatization and emotional consequences such as depression (43). It can even affect their social and cognitive capacity to respond to a new stressor. Evidence suggests that exacerbation of social isolation can be a consequence of overlapping previous stigma with a new one (38). In this case, previous stigma and social exclusion related to aging intersect the stigma related to COVID-19 infection. The elderly's reactions are highly influenced by the created social atmosphere (40), which in the case of COVID-19, has caused further isolation. Also, deficiencies in health care policies for older adults, especially in terms of mental health, have been a source of stigma (40, 44). The same cycle of misleading information, lack of support, and isolation happens here in the face of the COVID-19 outbreak. On the other side, young people seem to avoid social distancing or staying at home, as a way of denying their vulnerability and being at risk (45) and as a mechanism of putting the elderly in the “them” group who are at risk, while categorizing themselves in the “us” group that is strong and not at risk.

### Quarantine and Physical Distancing

Health experts believe that currently, the most effective way to control COVID-19 is to quarantine or maintain physical distance. How to effectively exercise these measures, however, is controversial. Even applying the term “Physical distancing” or “Social distancing” seems to be controversial, as the former refers to keeping a two-meter distance “physically” apart from others while maintaining the necessary social interactions via different methods such as using social media. The latter, however, might induce a sense of social isolation in the long term (46). Although some degree of physical distancing seems to be necessary, related isolation can lead to stigmatization and can be quite misleading. Quarantine and social isolation are often associated with poorer mental health outcomes and increased stigma (47, 48).

When, how long, to what extent, and who should be quarantined are critical questions that should be answered carefully and precisely. We mention here an extreme case of applying quarantine probably not based on scientific measures but as a measure of racial subjugation. During the plague pandemic in South Africa in 1901, evacuation of a whole ethnic group with forced arms took place without any evidence of exposure or scientific background (49). This is an example in which acting proactively against an infectious source blurred with preexisting racial disparities which resulted in an ethnically violent intervention. This is a lesson on the importance of applying careful consideration to recognizing which act is purely scientific and which one is influenced by preexisting social structures that can originate from or contribute to stigma.

## Consequences of Stigma

### Fear and Social Disruption

Lessens from previous pandemics such as HIV show us that fear of being stigmatized, along with misbeliefs about the disease, can be a barrier in seeking and receiving treatment (1, 25). There is a report about patients suspected to be infected by COVID-19 escaping from a hospital in Afghanistan. “Why the individuals left the medical facility was not immediately clear, but videos on social media suggested they were at odds with the hospital over their treatment.” (36–38, 46, 50). The blaming model of stigma proposes that people use several defense mechanisms to reduce the tension and anxiety related to the stigma of the disease, which includes either mature forms such as altruism and humor or immature ones such as denial and splitting (51, 52). Although assessing all sociocultural aspects of this multifaceted phenomenon is beyond the scope of this discussion, miscommunication, fear of social stigma, and defense mechanisms such as denial are playing an overt part.

This is not the first time that fear has overridden the risk of the disease. Back in 1901, in the US, during an epidemy of smallpox, several Italian immigrants destroyed a hospital that was built in their neighborhood for isolating people with smallpox. The social reactions were diverse but many newspapers called them “mob, unfit for autonomous governance” (53). Jumping to a conclusion and inferring the cause of the reaction to the nationality, ethnic background, literacy, and other immediate factors might be easy to reduce immediate tension but, is not an efficient and organized response. Such labeling obfuscates the real reasons; that is why and how people consider some medical services, such as quarantine and physical distancing, threatening situations, and act against them. Condemning citizens and showing in the media that people are acting irresponsibly and are not capable of a rational reaction to the threat seems to be useless and can exacerbate the tension (53). One reason for people's enactments might be that the emergency and extensiveness of the situation often hamper the effective communications of policymakers and health care professionals with general population. In these times, clarity, along with reassurance and communicating scientific information in simple words, is crucial. Both confusion and misunderstandings, and the presentation of “false science” by sources deemed to be thrust worthy, are breeding grounds of stigma as they evoke stereotypes, discriminatory behaviors, and prejudice. On the other hand, the impact of cultural beliefs and socially accepted norms cannot be overestimated (17, 54). Some cultural or traditional perspectives are skeptical of science and modern medicine. Even an untrusting government can play a role. Interpreting complex social responses like these is not easy and goes beyond the scope of this article, but it is worth mentioning that scapegoating, labeling, and stigmatizing can further complicate the situation.

### Health Outcomes

Experiencing different levels of emotional distress is indisputable in general populations, and this is further increased in affected patients, their caretakers, their family members, as well as the medical staff (55). The health consequences of stigma, however, go far beyond these senses. Stigma worsens physical and mental health outcomes. Stigma related to HIV, for instance, is associated with depression, anxiety, emotional and mental distress, and reduced quality of life. It also decreases the rate of adherence to treatment and access to medical facilities (12, 56). The fear of getting stigmatized causes people to avoid getting diagnostic tests (10, 56) and to reduce compliance with self-isolation rules and guidelines. Lack of accurate information, fear of judgment, and being discriminated against can lead to subconscious denial, therefore preventing being tested and refusing preventive strategies and treatment. Although increasing knowledge is a crucial measure, it is not enough for behavior alteration (11).

The stigmatized population is distrustful of the health care staff or authorities and resists cooperation in the event of a social health emergency. Stigma leads to a social misunderstanding of risk and extreme fear amongst members of society; that is accompanied by the disproportionate allocation of health care resources by politicians and health professionals (23). During the COVID-19 pandemic, long periods of quarantine, fear of illness, despair, fatigue, lack of life and personal protective equipment, insufficient and inconsistent information, financial issues, loss of loved ones, and stigma have been identified as factors which have influenced each other and are related to health outcomes (5).

Health care providers, on the other hand, are at a great risk of being stigmatized (55, 57). There are reports from around the world that doctors and other health care providers have been isolated from loved ones because of anticipated risk of contamination and assaulted physically or emotionally due to fear and stigmatization (55). This makes this already tough situation even more challenging as the increased burden on medical staff's mental health may negatively affect their functioning and resilience (3).

Anticipating the stigma related to COVID-19 health outcomes is essential to planning protective measures and affects both patients and health care providers. Stigma should be addressed rigorously as it can complicate and worsen the outcome, which necessitates careful planning and considerations and postulates more in-depth studies in this area.

## What Needs to Be Done?

Factors that cause stigma can be divided into three categories: predisposing (facilitators), precipitating (triggers), and perpetuating factors. Factors such as social structures and policies can increase stigma both as a predisposing and perpetuating factor, and require long-term planning. Factors such as insufficient information or contradictory messaging are precipitating factors and can be managed with immediate strategies that will be discussed here.

Predicting stigma related consequences of COVID-19 is essential in planning protective measures. Experiencing different levels of emotional distress is indisputable in general populations and is further increased in affected people. Patients' isolation and quarantine are effective measures, but they can increase stigma and severely impact on mental health and the economy. Isolation, loss of jobs, and financial burden, among other factors, can increase the risk of depression, especially in at-risk populations (47, 48, 58). Active screening and intervention, either through telephone, online communication such as video calling, or in-person, is essential in these situations.

To approach Covid-19 related stigma, one of the most important steps is to call it out. Lessons learned from HIV related stigma show that launching and supporting anti stigmatizing campaigns, adapting unifying symbols, and encouraging community activities are effective measures (1). The most famous symbol noticeable all around the world is the red ribbon, which is a symbol of HIV/AIDS awareness, care, empathy, and support. These measure also should include all people and how protective measures can adopt to the needs of people with especial needs. For instance, sunflower lanyard (hidden disabilities lanyard), prevents people who aren't able to wear face coverings from being isolated and stigmatized as not being compliant with the rules or designing lucent masks for people with hearing difficulties.

### Communication of Science

“*Despite the great discoveries and advances of science and medicine, primitive reactions to being confronted with disease continue to divide people and communities into ‘them’ and ‘us’”*.

Writes Gilmore in an article on HIV and stigma in 1994 (1). After almost 25 years, however, this is still the case. Although every literature on stigma suggests avoiding language and metaphors that polarize society, often the first reactions of authorities, hastily, is full of stigmatizing language.

The choice of language and metaphors is critical in de-stigmatizing efforts (1, 46, 59). It directs individuals and communities' reactions to obscure situations such as pandemics. Lessons learned from managing stigma in different infectious disease outbreaks suggest that military metaphors, such as fighting or combating COVID-19, can increase tension by inducing that there is an enemy in the society which everyone should fight (1, 60, 61). These metaphors even have been associated with self-willed death, such as suicidal ideations or requesting euthanasia in more chronic infections such as HIV (1).

Messaging seems to be the key. Conflicting messages from authorities, as well as misleading information from media, lead to fear and stigma (60). Pandemics provoke panic and anxiety due to their association with death and lack of certainty about the future. Encouraging empathy, altruism, and sublimation, along with focusing on human rights and respect, is essential (1). Promoting the concept that we are in this together “whether infected or affected by it” is critical to avoid polarization (1). Separating moral judgments and blame from physical avoidance and replacing it with empathy and care, as well as providing hope and an outlook to a brighter future, seems essential (21).

Although social media can be one of the most important sources of communication to eliminate feeling of loneliness and isolation during quarantine and periods of physical distancing, the downsides should be addressed carefully and step by step. Sending information through the influx of short and often non-transparent or even contradictory messages plays a role in increasing the anxiety, fear, and stigma felt in society. The sharing of inaccurate, nonscientific, and misleading information leads to further confusion and chaos. Therefore, proper training about using these networks and how to identify the source of messages, as well as increasing people's ability to distinguish between legitimate vs. nonsense messages, is recommended. These efforts can be done through school programs, health communities, as well as online public education, which requires both immediate and long-term interventions. Modifying misinformation and revising some mottos- especially the ones with military language that induce the sense of fighting response toward a foreign enemy- is necessary (46, 60, 61). Clarity of information and guidelines is the key to avoid confusion, fear, and the proceeding stigma. Measures like hotline services for counseling and public education can reduce the harmful mental health consequences of pandemics as is the case with COVID-19 (62) and are also helpful in reducing stigma.

Designing and implementing strategies is important, but constant evaluation of strategies and feedback is also crucial. Health care providers must actively reach out to people in society. While telemedicine is currently used extensively in the face of COVID-19, tele-follow up strategies should be considered too, while many people might be unable to be reached in person (58, 63). This necessitates both immediate and long-term planning, depending on the current infrastructures of each country.

Supporting structures and empowering people to take necessary measures by their own choice, focusing on altruism and responsibility, and building social trust is useful (47). In pandemics such as SARS and HIV, personal resources have been associated with decreased stigmatization as they positively affect rational risk estimation and rational response (30). Empathy and validating the experiences of the patients and their families through psychoeducation is important. For instance, generalizing and normalizing the grief experience as a common and accepted reaction to distress, and training management strategies such as talking and active listening are recommended. People should not be ashamed of talking about their personal experiences. Being supportive, understanding, and informative instead of blaming is helpful. Blaming and scapegoating people can cause prejudice and unnecessary guilt feelings, which increase stigma and non-compliance with public health directives (26).

A multidisciplinary workforce is essential. It is recommended that mental health assessment and treatment strategies get integrated into essential care needed for COVID-19 infected patients, their caregivers, and close relatives both in the hospital setting and community (19, 62). This should be addressed both by local treatment centers and policymakers to facilitate this integration by planning beneficial strategies of screening, diagnosing, referring, and treating psychiatric comorbidities (27). Currently, mental health providers have encountered an overwhelming challenge due to insufficient resources, lack of appropriate guidelines, and insufficient training in dealing with mental health consequences in the new situations due to COVID-19 (62). Guidelines and protocols of stigma reduction in health facilities and community centers are necessary. One of these guidelines has been provided by the WHO for public information and can be accessed through its website (57–64). Insights on the potential existence of double stigma and addressing it concurrently are vital.

In the global scope, pandemics have to be approached through international cooperation, which should replace localized and isolated strategies and mutual blaming. Empowering international organizations and improving collaborations are necessary measures (65). In the national scope, improving public confidence in health authorities can reduce stigma (23). This necessitates long-term planning and hard work to improve health care infrastructures accessible to every level of society. With the close collaboration of governments, health care providers, media, and communities to increase empathy and care and address misbeliefs and misinformation with accurately chosen terms and metaphors, the stigma related to COVID-19 will become manageable.

## Conclusion

Stigma is a barrier to medical evaluation, communication, delivering and receiving necessary care due to fear and is associated with both physical and mental health complications. COVID-19 related stigma needs to be addressed rigorously by professionals and health care providers as well as authorities. Gathering more specific information on its different facets seems urgent. Lessons learned from previous pandemics show that multidimensional approaches for health care, considering all bio-psycho-socio aspects and employing strategies to enhance communities' empathy and resilience, pave the way toward the goal of reducing stigma. Clarity of information and guidelines, as well as continuous screening of speech pitfalls and inappropriate metaphors, is suggested. This in turn will lead to a stronger sense of unity, more effective scientific communication, increased compliance with rules and guidelines set out to battle the pandemic, more efficient use of medical pathways and eventually a better management of the pandemic as a whole.

## Author Contributions

FS, RM, SZM, FB, RA, and SBM had considerable contributions to acquisition and interpretation of documents and drafting the manuscript. SBM and RR had substantial contribution to the conception and revising the manuscript critically. All the authors have approved the submitted version and agreed to be accountable for all aspects of the work. All authors contributed to the article and approved the submitted version.

## Conflict of Interest

The authors declare that the research was conducted in the absence of any commercial or financial relationships that could be construed as a potential conflict of interest.
